# Real-time imaging of surface chemical reactions by electrochemical photothermal reflectance microscopy†

**DOI:** 10.1039/d0sc05132b

**Published:** 2020-12-15

**Authors:** Cheng Zong, Chi Zhang, Peng Lin, Jiaze Yin, Yeran Bai, Haonan Lin, Bin Ren, Ji-Xin Cheng

**Affiliations:** Department of Biomedical Engineering, Department of Electrical & Computer Engineering, Department of Chemistry, Department of Physics, Photonics Center, Boston University Boston MA 02215 USA jxcheng@bu.edu; State Key Laboratory of Physical Chemistry of Solid Surfaces, Collaborative Innovation Center of Chemistry for Energy Materials, College of Chemistry and Chemical Engineering, Xiamen University Xiamen 361005 China bren@xmu.edu.cn

## Abstract

Traditional electrochemical measurements based on either current or potential responses only present the average contribution of an entire electrode's surface. Here, we present an electrochemical photothermal reflectance microscope (EPRM) in which a potential-dependent nonlinear photothermal signal is exploited to map an electrochemical process with sub-micron spatial resolution. By using EPRM, we are able to monitor the photothermal signal of a Pt electrode during the electrochemical reaction at an imaging speed of 0.3 s per frame. The potential-dependent photothermal signal, which is sensitive to the free electron density, clearly revealed the evolution of surface species on the Pt surface. Our results agreed well with the reported spectroelectrochemical techniques under similar conditions but with a much faster imaging speed. We further mapped the potential oscillation during the oxidation of formic acid on the Pt surface. The photothermal images from the Pt electrode well matched the potential change. This technique opens new prospects for real-time imaging of surface chemical reaction to reveal the heterogeneity of electrochemical reactivity, which enables broad applications to the study of catalysis, energy storage, and light harvest systems.

## Abstract 2

The potential-dependent photothermal signal, which is sensitive to the free electron density, map the evolution of surface species on the electrode in real time.

## Graphical abstract

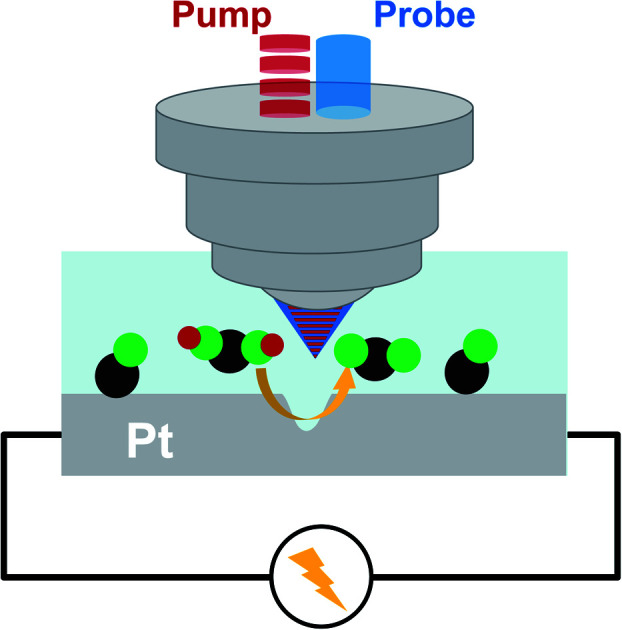

## Introduction

The electrochemical interface is a highly dynamic and heterogeneous region where electron transfer, energy conversion and storage, and mass exchange phenomena occur. Typical electrochemical methods based on either current or potential responses only reflect the average contribution of an entire electrode surface. However, even well-prepared electrode surfaces contain defects which could bring about differences in the electrochemical activities. Thus, a detailed characterization of the heterogeneity of electrochemical interfaces is essential for the applications that require a precise characterization of the electrode surfaces, for solar cells, electrocatalytic systems, energy storage devices, light harvesting apparatus, *etc.*^1^

Advanced microscopic methods have been developed to probe the local electrochemical processes occurring at the interface with high spatial resolution. Of them, scanning electrochemical microscopy is the most popular electrochemical imaging method for unravelling the relationship between electrochemical properties and the local structural features of a surface.^2,3^ However, the imaging speed of traditional scanning electrochemical microscopy ranges from a few minutes to a few hours per frame, due to the move-stop-measure scan mode and a trade-off between probe response time and current detection sensitivity.^4^*In situ* spectroscopic methods such as electroreflectance,^5–9^ infrared spectroscopy,^10,11^ Raman spectroscopy,^12^ sum-frequency generation spectroscopy,^13,14^ second harmonic generation,^15^ and their surface-enhanced derivatives need relative long signal integration time for each measured spot. To real-time monitor the complete electrochemical processes, including the intermediate species, on the whole surface, an electrochemical imaging approach with a temporal resolution compatible with the process on the electrode surface is required.

Here, we report an electrochemical imaging method in which a potential-dependent nonlinear photothermal signal is used to monitor space- and time-resolved electrochemical processes. Photothermal reflectance microscope indirectly measures the absorption and allows for label-free imaging of single nonfluorescent molecules.^16,17^ Our photothermal microscopy is based on the detection of laser-induced temperature-dependent variations of the refractive index through the intensity change of a probe beam in the local environment of an absorbing species.^18^ In general, the photomodulated optical reflectance is dependent on two contributing mechanisms: thermal and Drude (free-carrier) modulation:Δ*R* = (∂*R*/∂*T*)Δ*T* + (∂*R*/∂*N*)Δ*N*where, ∂*R*/∂*T* is the temperature reflectance coefficient, and ∂*R*/∂*N* is the Drude reflectance coefficient. Δ*T* is the modulated sample temperature, and Δ*N* is the modulated free-carrier density.^19,20^ The first part of the equation is the underlying mechanism of conventional photothermal microscopy. The second part of the equation shows that Δ*R* is, in general, linear with the free electron density. The Δ*R* often displays exceedingly high sensitivity to changes on the electrode surface, such as those induced by changes of charge on the interface, or adsorption of species from the electrolyte.^5,6^

Unlike the transverse light path in photothermal deflection spectroscopy,^21,22^ we employed a collinear light path design to achieve high-resolution microscopic imaging. Our electrochemical photothermal reflectance microscope (EPRM) synchronized a nonlinear photothermal imaging microscope with transient electrochemical methods like cyclic voltammetry (CV) and chronopotential method. First, we exploit EPRM to monitor the formic acid electro-oxidation on a Pt electrode in an acidic solution. This method explicitly presents the heterogeneity of a Pt electrode during the electrochemical reaction. Then, we demonstrate the ion adsorption sensitivity of EPRM in monitoring the CV process of the Pt electrode in H_2_SO_4_ solution. Finally, we report EPRM mapping of potential oscillation in a galvanostatic electrooxidation of formic acid on a Pt surface.

## Method and materials

**Fig. 1:**
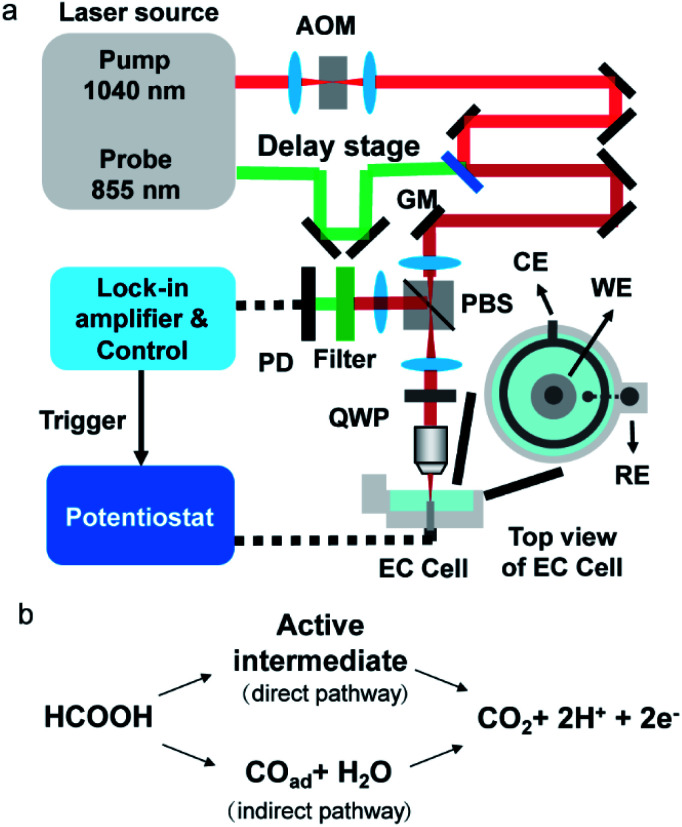
(a) Schematic of the electrochemical photothermal reflectance microscope. AOM: acousto-optic modulator; GM: galvo mirror; PBS: polarizing beam splitter; QWP: quarter waveplate; PD: photodiode; WE: working electrode; CE: counter electrode; RE: reference electrode. (b) Scheme of the dual path mechanism of the formic acid oxidation.

A schematic of our setup is depicted in Fig. 1a. A femtosecond laser source (InSight DS+, Spectral Physics) outputted 1040 nm pump laser and 855 nm probe laser. The pump beam was modulated by an acousto-optic modulator at 2.3 MHz. The time delay between the pump and probe lasers was set as 20 ps by a translational stage to avoid generating the transient adsorption signal and stimulated Raman signal. The 60 mW pump and the 10 mW probe beams were spatially aligned and sent to a lab-built laser scanning upright microscope (X51, Olympus). The scanned beams passed through a quarter-waveplate and were focused on an electrode surface by a 40× water immersing objective (N.A. 0.8, Olympus). Double-pass of the quarter waveplate changed the polarization of the back-reflected beams. A polarizing beam splitter was placed before the quarter-waveplate to separate the back-reflected laser from excitation laser and allowed forward light to pass through and the output signals to reflect. The output beam was filtered by a bandpass filter to remove the pump beam composition and was directed to a photodiode with a lab-built resonant amplifier. To improve the collection efficiency, we installed a collection lens before the photodiode. A lock-in amplifier was used to demodulate the photothermal signal. The photothermal imaging was performed at a speed of 10 μs per pixel. Each photothermal image contains 200 × 200 pixels (1 pixel = 150 nm), resulting in 0.6 s per frame. The CV curve and chronopotential curve were simultaneously recorded by a potentiostat (CHI 1240c) for CV processes and a potentiostat (XMU QJ2800) for the galvanostatic mode. Before photothermal imaging, a TTL signal from the control was sent to the potentiostat to initiate the potential sweep. The exposure time for every frame was 0.6 s which was the same as the time interval of the electrochemistry method. Therefore, the electrochemical signal (*i.e.*, current and potential) and imaging data were synchronized. The polycrystalline Pt electrode (diameter = 2 mm, geometric area = 3.14 × 10^−2^ cm^2^) was mechanically polished by 1 μm Al_2_O_3_ powder. After ultrasonic cleaning in ultrapure water (18.2 MΩ cm), the electrode was transferred to a home-built spectroelectrochemical cell and was cleaned in 0.5 M H_2_SO_4_ by several potential cycles from −0.65 V to 0.4 V before the measurement (Fig. S1†). The same Pt electrode by the same polishing method was used in all experiments. The typical electrochemical surface area, by calculating the hydrogen adsorption wave,^23^ is 4.4 × 10^−2^ cm^2^ to 4.8 × 10^−2^ cm^2^. The surface roughness, the ratio between electrochemically active surface area and geometric area, is about 1.4 to 1.5. All electrode potentials were referred to the mercury sulfate electrode (MSE).

## Results and discussion

### Imaging electrocatalytic process of formic acid on Pt surface

Formic acid is an attractive chemical fuel for fuel cell applications due to its high energy density and is an ideal model molecule for the study of electrocatalysis.^24^ Among all pure metals, Pt exhibits the highest activity toward the electro-oxidation of formic acid. The formic acid oxidation on a Pt surface is well known to proceed with a dual path mechanism (direct and indirect pathway, Fig. 1b).^24^

**Fig. 2:**
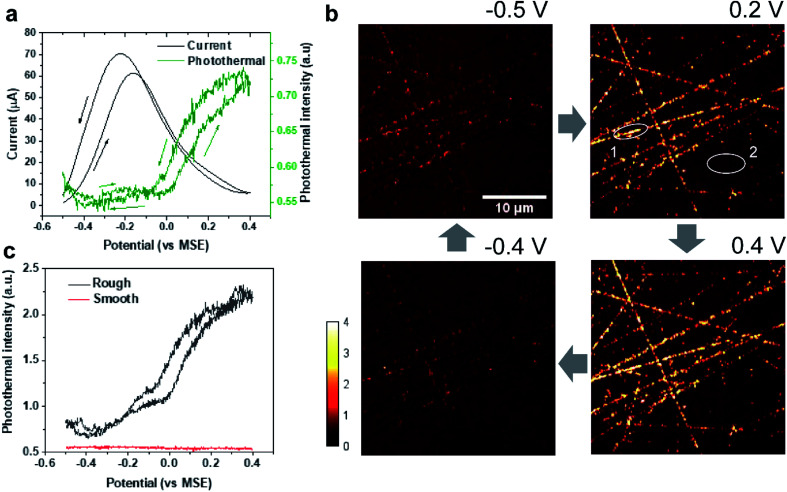
EPRM monitoring of the electrocatalytic oxidation of formic acid on Pt surface. (a) CV curve (black) of the Pt electrode and corresponding average photothermal intensity (green) of the whole imaging area as shown in (b) during CV measurement in 1 M HCOOH in a 0.5 M H_2_SO_4_ solution with a scan rate of 3 mV s^−1^. The arrows indicate the potential sweep direction. (b) Photothermal images of the Pt electrode obtained at different potentials during electrocatalytic oxidation of formic acid. The blue arrows indicate the potential sequence. (c) Local potential-dependent photothermal intensity in rough (black, spot 1 in (b)) and smooth areas (red, spot 2 in (b)).

Here, EPRM was demonstrated by synchronized photothermal imaging and CV measurement of 1 M formic acid in a 0.5 M H_2_SO_4_ solution at a scan rate of 3 mV s^−1^ (Movie 1†). With a commonly used potential sample interval of 2 mV, the time interval of the potential step is *ca.* 667 ms, matching the speed of photothermal imaging. As shown in Fig. 2a, the CV shows a positive sweeping peak at −0.16 V corresponding to the decomposition of formic acid and the oxidation of the adsorbed CO and a negative sweeping peak at −0.22 V due to the activation of surface sites. Because the thin electrolyte layer in the spectroelectrochemical cell results in the inhibition of mass transfer and the slow scan rate,^25^ the second anodic peak at *ca.* 0.2 V is not very clear (as shown in Fig. S2†). The photothermal intensity of Pt electrode is averaged over the entire image area and plotted against the electrode potential, like a CV curve. As shown in Fig. 2a, the formic acid oxidation on the Pt electrode can be divided into two stages *via* the trend of photothermal intensity: (1) from −0.5 V to 0 V, where the photothermal intensity is low and independent of potential, and (2) from 0 V to 0.4 V, where the photothermal intensity increases with the positive-going potential sweep. This phenomenon can be explained by the CO adsorption/oxidation on the Pt electrode and the oxidation of the Pt surface.^8^ At the beginning of the potential sweep, a part of formic acid is dissociated to form adsorbed CO (CO_ad_) on the Pt surface. In this case, the CO to Pt forward donation of electron greatly outweighs the back-donation, the net charge of the Pt is negative, which leads to a high free electron density of metal, large reflectance of the probe light and low photothermal intensity (Fig. 2b, −0.5 V).^26^ With the positive potential sweep, the photothermal signal starts to increase at 0 V due to the oxidation of CO to CO_2_ with adsorbed OH (Fig. 2b, 0.2 V and 0.4 V). This result agrees well with previous surface-enhanced infrared spectroscopic^10^ and electroreflectance results.^7,27^ On the reversed negative potential sweep, the photothermal intensity decreases gradually to the initial intensity level at −0.1 V and scarcely changed from −0.1 V to −0.5 V. Our results indicate that EPRM can map the reflectance change of a Pt electrode interface during the electrochemical process in real-time and reveal the evolution of a catalyst poison (CO) during the electrocatalysis reaction.

**Fig. 3:**
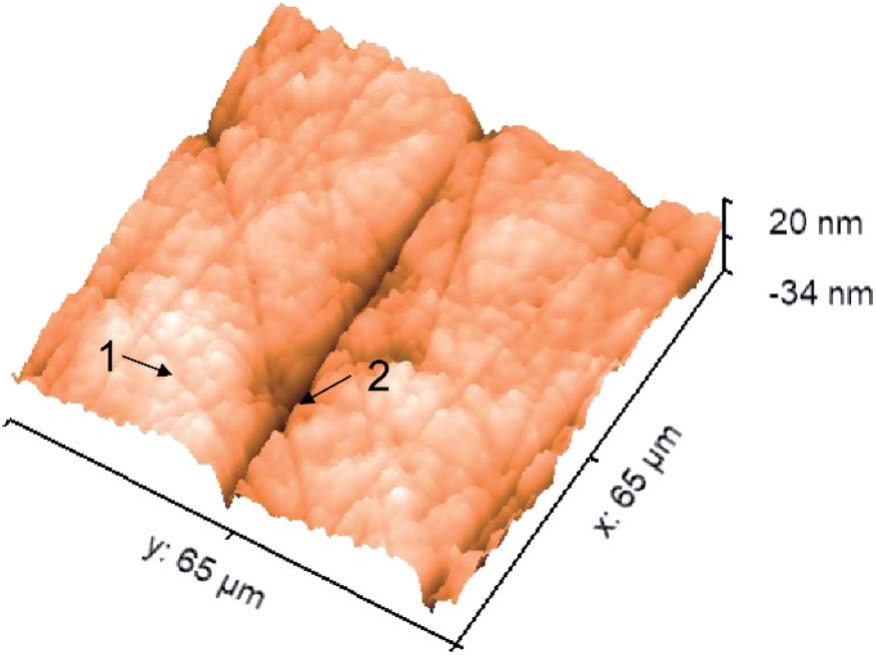
Typical local topography of a polished Pt electrode. Spot 1: a smooth area; spot 2: a rough area (a scratch).

Importantly, with 500 nm spatial resolution (as shown in Fig. S3†), EPRM demonstrates the heterogeneity of the Pt surface, as shown in Fig. 2b. At the beginning of the potential cycle (−0.5 V), the Pt electrode presents a uniform surface with sporadic “hot spots”. With a positive sweep of the potential to 0.2 V, the rough feature which originates from the mechanical polishing shows a much stronger photothermal signal than the flat area. The maximum difference between rough and smooth regions appears at 0.4 V. With a negative sweep of the potential back to −0.4 V, the surface returns to the homogeneity state. Fig. 2c clearly shows the huge difference in the photothermal signal of rough and smooth areas. Using a white light interferometric profiler (Zygo), we measured the local topography of a polished Pt electrode surface, as shown in Fig. 3. The standard deviation of the height of a smooth area (Fig. 3, spot 1) is about 2 nm and the depth of a rough area (*e.g.*Fig. 3, spot 2) is about 20 to 30 nm. Comparing the topography of the Pt electrode and its corresponding photothermal image (Fig. S4†), the photothermal intensity from a rough area is much higher than that from a smooth region, resulting from the larger adsorption cross-section of nanostructures in the rough area. On the other hand, the potential-dependent photothermal signal in rough areas exhibits a more notable change than that in smooth areas and the averaged of the photothermal signal. This could be due to the relatively larger surface area, hence more adsorbed molecules in rough areas than that in flat regions, which could lead to a more significant intensity change with potential. Another possible reason is that the electrochemical activity is higher in rough areas. Our results demonstrate the potential of EPRM for mapping inhomogeneous electrochemical properties on the surface, which may be overlooked by using the average signal alone.

### Monitoring ion adsorption on the Pt electrode

**Fig. 4:**
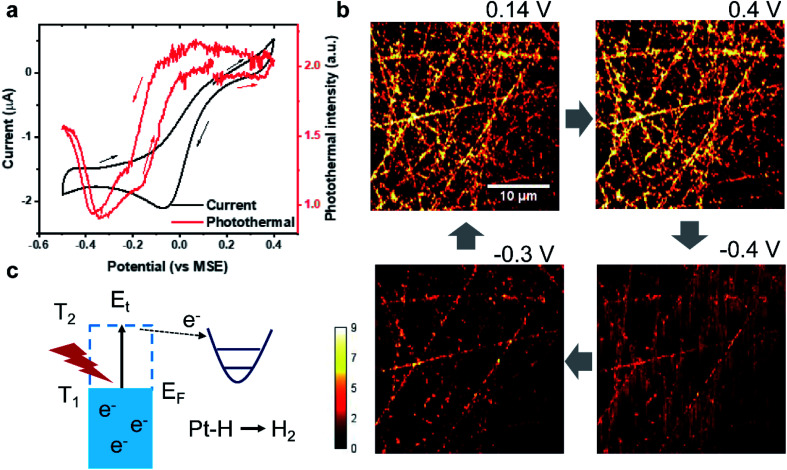
EPRM monitoring during the CV of Pt in H_2_SO_4_ solution. (a) CV curve (black) and corresponding average photothermal intensity (red) during CV measurement of the Pt electrode in a 0.5 M H_2_SO_4_ solution at a scan rate of 3 mV s^−1^. The arrows indicate the potential sweep direction. (b) Photothermal images of the Pt electrode at different potentials. The blue arrows indicate the potential sequence. (c) Scheme of the photothermal-induced hydrogen generation process.

To further prove the surface species sensitivity of EPRM, we studied the potential dependent photothermal signal from the Pt electrode in sulfuric acid without formic acid. Fig. 4a shows the CV and the average photothermal signal from a Pt electrode immersed in a 0.5 M H_2_SO_4_ solution at a scan rate of 3 mV s^−1^ (Movie 2†). The dwell time of potential step and the imaging time per frame are both *ca.* 667 ms.

At first, the potential was swept from open circuit potential (0.14 V) to 0.4 V, then reversed to −0.5 V, and finally returned to 0.14 V. The CV shows a clear reduction peak at −0.05 V and a huge background current due to the reduction process of dissolved oxygen at a slow scan rate, as shown in Fig. 4a. The Pt surface has three different chemical states during the CV process: (1) the oxide region where a monolayer of oxide species exists on the surface (0 V to 0.4 V), (2) the double-layer region (−0.3 V to 0 V), and (3) the hydrogen adsorption region where a monolayer of reversibly adsorbed atomic hydrogen covers the surface (−0.5 V to −0.3 V). In the oxide region, the photothermal signals almost remain constant (Fig. 4b, 0.14 V and 0.4 V). In the double-layer region, the photothermal intensity decreases with the negative potential scanning (Fig. 4b, −0.3 V). We attribute the decreased photothermal signal to the modification of the surface by the bisulfate adsorption on the Pt surface. The binding energy of Pt 5d is close to the LUMO of bisulfate. Hence, the back donation (charge transfer from the Pt surface to the bisulfate) plays a major role.^28^ From 0 V to −0.3 V, the coverage of bisulfate decreases, thus free electron density of Pt increases. We find a more interesting result that the photothermal curve agrees well with the bisulfate coverage from previous auger electron spectroscopy measurements, radioactive labelling method, and infrared results.^28–30^ To further verify our assumption, we measured the potential-dependent photothermal signal in H_2_SO_4_ after removing dissolved oxygen by fluxing nitrogen gas for 2 hours. Fig. S5† presents the potential-dependent photothermal signal in deoxygenated H_2_SO_4_ solution. We note that O_2_ from air could return to our spectroelectrochemical cell during the experiment even the cell was sealed. Yet, the photothermal *vs.* potential curves in H_2_SO_4_ with dissolved O_2_ (Fig. S5a†) and in H_2_SO_4_ with O_2_ partial (>50%) removed (Fig. S5b†) are almost identical. In accordance, it was shown that the Faradaic reaction of dissolved O_2_ does not affect the reflectance of the Pt surface.^31^ In addition, we measured the potential-dependent photothermal signal in HClO_4_ as a control experiment. Due to a weak affinity of perchlorate on a Pt surface,^32^ the potential-dependent photothermal curve (Fig. S6†) shows a near-linear relationship with the potential change from −0.4 V to 0.4 V. This result indicates the bisulfate ion adsorption is responsible for the different observations in sulfuric and perchloric acid solutions.

In the hydrogen adsorption region, we found an interesting phenomenon. The average photothermal signal increases with a negative potential scan, while the image displays vertical lines which started from rough areas and followed the laser scanning trajectory. The reversible peaks at −0.4 V correspond to the strong adsorption hydrogen on Pt surface. This abnormal image (Fig. 4b, at −0.4 V) might result from the following factors: (1) the adsorbed hydrogen on the surface, and (2) rough areas. This phenomenon only happens in the potential range of hydrogen adsorption and does not appear in the experiment of formic acid at −0.4 V (Fig. 2b), since the hydrogen adsorption is suppressed by the adsorption of CO in the HCOOH/H_2_SO_4_ solution. In addition, the vertical lines all started in rough areas. Therefore, we assumed that this phenomenon is the consequence of the photothermal induced hydrogen evolution reaction as shown in Fig. 4c. Given the nanostructures in rough areas, the strong photothermal effect increases the local temperature which consequently reduces local potential and triggers the generation of H_2_ nanobubbles.^33–35^ The generation of hydrogen bubbles significantly impacts the local reflectivity. After passing the rough areas, the hydrogen nanobubbles are trapped by the scanning laser for a short duration, contributing to the observed vertical lines.

In addition, we compared the potential-dependent photothermal signals in H_2_SO_4_ solution (Fig. 4a) and the HCOOH/H_2_SO_4_ solution (Fig. 2a). The photothermal *vs.* potential curves are quite different because of the CO adsorption and oxidation. The intensity of photothermal signal obtained from the two systems are related to the various degrees of roughness.

### Mapping the potential oscillation of formic acid on Pt surface

To further demonstrate EPRM for study the dynamic process, we mapped the potential oscillation in the oxidation of formic acid on a Pt surface. By decreasing pixel dwell time to 5 μs, the imaging time per frame was achieved at *ca.* 330 ms. The oscillation was generated by the oxidation of 1 M HCOOH in a 0.5 M H_2_SO_4_, where the applied current was 20 μA.

**Fig. 5:**
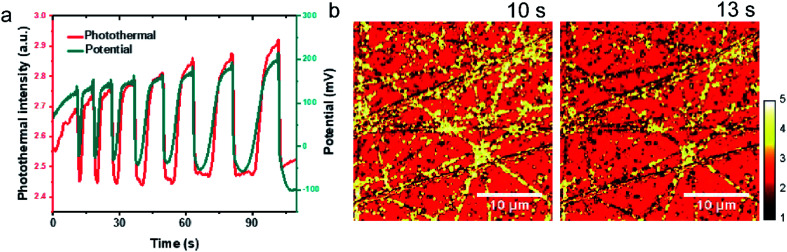
EPRM monitoring the potential oscillation during the oxidation of 1 M HCOOH of Pt in H_2_SO_4_ solution. (a) The potential oscillations observed in the oxidation of formic acid at a constant current at 20 μA in 1 M HCOOH + 0.5 M H_2_SO_4_ and corresponding photothermal signal. (b) Typical photothermal images of Pt surface at different time.

As shown in Fig. 5a, at the moment of the current application, it is an induction period of *ca.* 10 s where potential rises from 0.1 V to *ca.* 0.15 V. Then the potential exhibits a small sharp potential spike and then appears sharp oscillations with large dips to −0.05 V. The potential oscillator happens between *ca.* −0.1 V to *ca.* 0.2 V. The interval times between dips are steadily increased from 7.4 s to 19.1 s. As the mechanism of potential oscillation described in the previous paper,^36^ the galvanostatic potential oscillation occurs in the following way. When the electrode potential is low, elongated reaction time leads to the accumulation of CO at the electrode surface gradually and blocks the reactive sites, which raises the potential to maintain the applied current. When the potential becomes high enough, CO_ad_ reacts with adsorbed water to produce CO_2_ resulting in a potential drop and leading to the formation of CO. This cycle repeats itself to give the sustained temporal potential oscillations.

Movie 3† recorded the time-dependent photothermal images during the potential oscillation process. Typical time-lapse photothermal images are present in Fig. 5b. To find the correlation between potential oscillation and photothermal signal, the average photothermal signal of the whole image area is plotted as a red line in Fig. 5a as a function of time. The coincidence of the dips in the potential-time and photothermal-time plots ensures the synchronization of the electrochemical and photothermal measurements. The temporal change of the photothermal intensities well match with the potential oscillation. The photothermal signal increases at the small potential spikes (Fig. 5b, 10 s) and the photothermal intensity reaches its minimum when potential drops (Fig. 5b, 13 s). After the drop of the potential to the low limiting value, the photothermal signal recovers its intensity quickly and then gradually. As we discussed above, the photothermal signal could reflect the free carrier density. The high potential results in a low electron density on Pt surface and higher photothermal signal and *vice versa*. This result eloquently demonstrates that photothermal signal affords a means of monitoring interfacial potentials change with a very high time and spatial resolution.

### Power-dependence of photothermal intensity

**Fig. 6:**
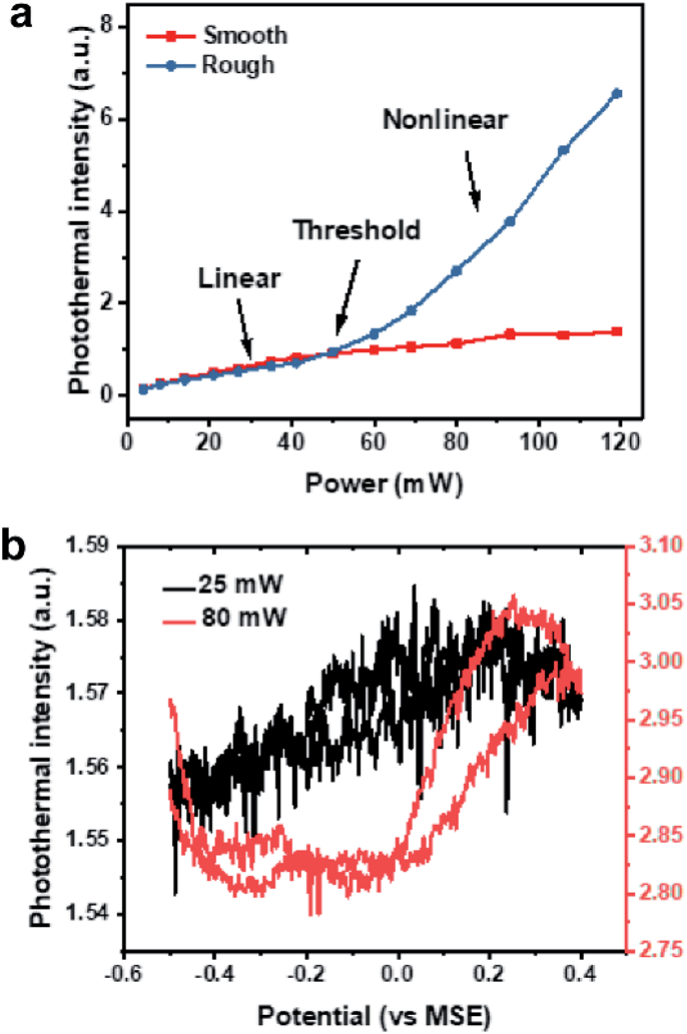
Power-dependence of photothermal intensity. (a) Photothermal intensity in rough and smooth areas as a function of pump laser power. (b) Potential dependent photothermal intensity in linear (pump power 25 mW, black) *versus* nonlinear range (pump power 80 mW, red) during of electrocatalytic oxidation of formic acid on Pt surface.

To verify our EPRM contrast mechanism, we studied the power-dependence of the photothermal signal. Here, all laser powers were measured before the microscope. As shown in Fig. S7,† the photothermal intensity of the Pt electrode shows a linear response to the probe laser power. In Fig. 6a, the photothermal signals in rough areas and smooth areas present different pump laser power dependence relationships. The photothermal signals in smooth areas are linear with power. The photothermal signals in rough areas exhibit a linear behaviour at a low power of pump laser and a near-quadratic behavior (Fig. S8†) when the power exceeds a threshold (*ca.* 50 mW). There are two possible reasons for this nonlinear photothermal signal: (1) associated with nanobubble formation around the overheated surface;^37,38^ (2) two-photon adsorption process. More interestingly, the potential-dependent photothermal signal is more evident in the nonlinear regime than in the linear regime, as shown in Fig. 6b. The maximum intensity change is 1.2% for linear EPRM and 9.3% for nonlinear EPRM. This result indicates that the nonlinear photothermal process amplifies the Drude effect of reflectance. The detailed enhancement mechanism still requires further study.

### Local temperature change during the photothermal detection

To estimate the temperature change during the photothermal measurement, we used COMSOL to simulate the temperature swing by periodic laser irradiation. The detailed simulation model can be found in the ESI 1.† As shown in Fig. S9,† the temperature of the illuminated Pt surface rises quickly and then gradually reaches 426 K. After the laser illumination is off, the surface temperature rapidly recovers to initial temperature (293 K). The local temperature spike might affect electrochemical reactivities to some extent, for example, early appearance of H_2_ nanobubbles before the hydrogen evolution potential. The local temperature of surface water also periodically rises and decays with the laser irradiation. The maximum temperature is 300 K and the final temperature is 297 K after 10 μs laser illumination. The simulation result indicates that the solution temperature change is negligible due to the high heat capacity of water.

## Conclusions

The current work demonstrated EPRM for the study of chemical reactions on metal electrodes. The potential-dependent photothermal signal, which is sensitive to the free electron density, clearly revealed the evolution of surface species on the Pt surface. When comparing the results of CV processes of Pt in sulfuric acid solution with and without formic acid, it is clear the CO adsorption and oxidation plays an important role in the change of photothermal signal. We further verified that the EPRM is sensitivity on the ion adsorption on the electrode. In addition, our method could monitor the galvanostatic potential oscillation of formic acid. Our method displays a high sensitivity to the electrochemical process in the electrode surface. In the future, this method could be extended to the mid-infrared region to further provide the fingerprint information of surface molecules during an electrochemical reaction.^39,40^ Collectively, this technique opens new prospects for imaging surface chemical reactions in real-time, which is promising for catalysis, energy storage devices, and light harvest applications.

## Conflicts of interest

There is no conflict to declare.

## Supplementary Material

SC-012-D0SC05132B-s001

SC-012-D0SC05132B-s002

SC-012-D0SC05132B-s003

SC-012-D0SC05132B-s004

## References

[cit1] Oja S. M., Fan Y., Armstrong C. M., Defnet P., Zhang B. (2015). Anal. Chem..

[cit2] Amemiya S., Bard A. J., Fan F.-R. F., Mirkin M. V., Unwin P. R. (2008). Annu. Rev. Anal. Chem..

[cit3] Polcari D., Dauphin-Ducharme P., Mauzeroll J. (2016). Chem. Rev..

[cit4] Kang M., Momotenko D., Page A., Perry D., Unwin P. R. (2016). Langmuir.

[cit5] Jebin Jacob Jebaraj A., Scherson D. (2014). Anal. Chem..

[cit6] Conway B., Angerstein-Kozlowska H., Laliberte L. (1974). J. Electrochem. Soc..

[cit7] Adzi R., Podlavicky M. (1977). J. Phys., Colloq..

[cit8] Onderwaater W. G., Taranovskyy A., van Baarle G. C., Frenken J. W., Groot I. M. (2017). J. Phys. Chem. C.

[cit9] Celebrano M., Sciascia C., Cerullo G., Zavelani-Rossi M., Lanzani G., Cabanillas-Gonzalez J. (2009). Adv. Funct. Mater..

[cit10] Samjeské G., Miki A., Ye S., Osawa M. (2006). J. Phys. Chem. B.

[cit11] Zhou Z.-Y., Tian N., Chen Y.-J., Chen S.-P., Sun S.-G. (2004). J. Electroanal. Chem..

[cit12] Wu D.-Y., Li J.-F., Ren B., Tian Z.-Q. (2008). Chem. Soc. Rev..

[cit13] Cimatu K., Baldelli S. (2006). J. Am. Chem. Soc..

[cit14] Schultz Z. D., Biggin M. E., White J. O., Gewirth A. A. (2004). Anal. Chem..

[cit15] Corn R. M., Higgins D. A. (1994). Chem. Rev..

[cit16] Gaiduk A., Yorulmaz M., Ruijgrok P., Orrit M. (2010). Science.

[cit17] Gaiduk A., Ruijgrok P. V., Yorulmaz M., Orrit M. (2010). Chem. Sci..

[cit18] Vermeulen P., Cognet L., Lounis B. (2014). J. Microsc..

[cit19] Wagner R. E., Mandelis A. (1996). Semicond. Sci. Technol..

[cit20] Wagner R. E., Mandelis A. (1996). Semicond. Sci. Technol..

[cit21] Deng Z., Spear J. D., Rudnicki J. D., McLarnon F. R., Cairns E. J. (1996). J. Electrochem. Soc..

[cit22] Pawliszyn J., Weber M. F., Dignam M. J., Park S. M. (1986). Anal. Chem..

[cit23] Wei C., Sun S., Mandler D., Wang X., Qiao S. Z., Xu Z. J. (2019). Chem. Soc. Rev..

[cit24] Boronat-González A., Herrero E., Feliu J. M. (2017). Curr. Opin. Electrochem..

[cit25] Okamoto H., Kon W., Mukouyama Y. (2005). J. Phys. Chem. B.

[cit26] Wong Y. T., Hoffmann R. (1991). J. Phys. Chem..

[cit27] Fromondi I., Scherson D. A. (2006). J. Phys. Chem. B.

[cit28] Thomas S., Sung Y.-E., Kim H., Wieckowski A. (1996). J. Phys. Chem..

[cit29] Zeng D.-M., Jiang Y.-X., Zhou Z.-Y., Su Z.-F., Sun S.-G. (2010). Electrochim. Acta.

[cit30] Kolics A., Wieckowski A. (2001). J. Phys. Chem. B.

[cit31] McIntyre J. D. E., Kolb D. M. (1970). Symp. Faraday Soc..

[cit32] Macia M. D., Campina J. M., Herrero E., Feliu J. M. (2004). J. Electroanal. Chem..

[cit33] Climent V., Coles B. A., Compton R. G., Feliu J. M. (2004). J. Electroanal. Chem..

[cit34] Ledezma-Yanez I., Wallace W. D. Z., Sebastián-Pascual P., Climent V., Feliu J. M., Koper M. T. (2017). Nat. Energy.

[cit35] Climent V., Coles B. A., Compton R. G. (2002). J. Phys. Chem. B.

[cit36] Naito M., Okamoto H., Tanaka N. (2000). Phys. Chem. Chem. Phys..

[cit37] Zharov V. P. (2011). Nat. Photonics.

[cit38] Nedosekin D. A., Galanzha E. I., Dervishi E., Biris A. S., Zharov V. P. (2014). Small.

[cit39] Zhang D., Li C., Zhang C., Slipchenko M. N., Eakins G., Cheng J.-X. (2016). Sci. Adv..

[cit40] Li C., Zhang D., Slipchenko M. N., Cheng J.-X. (2017). Anal. Chem..

