# Supplementary material for: Real-time imaging of surface chemical reactions by electrochemical photothermal reflectance microscopy
Source: Chem Sci. 2020 Dec 15;12(5):1930–6. doi: 10.1039/d0sc05132b (PMC8179047; doi:10.1039/d0sc05132b)
Supplement: SC-012-D0SC05132B-s001 [file SC-012-D0SC05132B-s001.pdf]

Electronic Supplementary Material (ESI) for Chemical Science.
This journal is © The Royal Society of Chemistry 2020

Supporting information of Real-time imaging of Surface Chemical Reactions by Electrochemical Photothermal Reflectance Microscopy

Cheng Zong[a,b], Chi Zhang[a], Peng Lin[a], Jiaze Yin[a], Yeran Bai[a], Haonan Lin[a], Bin Ren[*b], and Ji-Xin Cheng[*a]

a. Department of Biomedical Engineering, Department of Electrical & Computer Engineering, Department of Chemistry, Department of Physics, Photonics Center, Boston University, Boston, MA 02215, USA

b. State Key Laboratory of Physical Chemistry of Solid Surfaces, Collaborative Innovation Center of Chemistry for Energy Materials, College of Chemistry and Chemical Engineering, Xiamen University, Xiamen 361005, China

Correspondence to: bren@xmu.edu.cn, jxcheng@bu.edu

Figure S1. Cyclic voltammogram (CV) of Pt in H_2SO_4

Figure S2. Cyclic voltammogram of HCOOH in H_2SO_4

Figure S3. Spatial resolution of EPRM

Figure S4. Typical topography of Pt electrode and its corresponding photothermal image

Figure S5. The photothermal signal in deoxygenated H_2SO_4 solution.

Figure S6. The photothermal signal in $HClO_4$ solution.

Figure S7. The photothermal intensity vs the probe laser power.

Figure S8. The photothermal intensity vs the pump laser power.

Supporting information 1. Local temperature change at the Pt electrode

Movie 1：EPRM monitoring of the electrocatalytic oxidation of formic acid on Pt surface.

Movie 2：EPRM monitoring during the CV of Pt in H_2SO_4 solution.

Movie 3：EPRM monitoring the potential oscillation during the oxidation of 1 M HCOOH of Pt in H_2SO_4 solution.

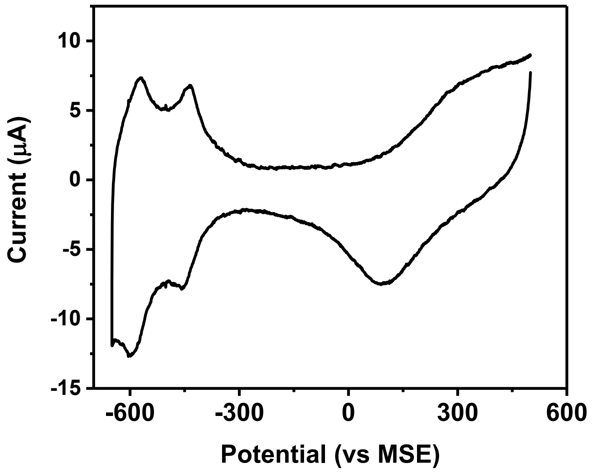

Figure S1 Cyclic voltammogram (CV) of a Pt electrode in 0.5 M H_2SO_4 at a scan rate of 100 mV/s. High scan rate can suppress the redox process of dissolved O_2

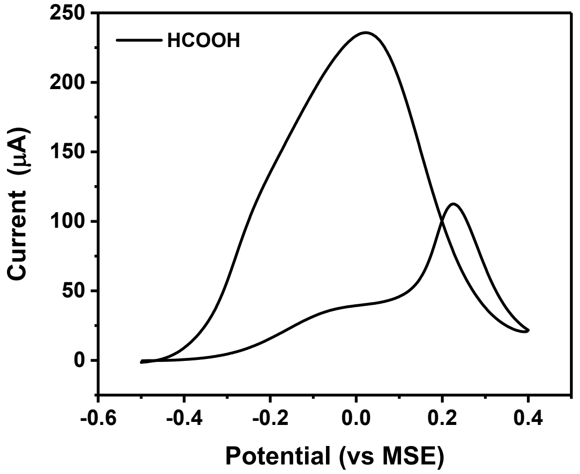

Figure S2 Cyclic voltammogram of a Pt electrode in 1 M HCOOH + 0.5 M H_2SO_4 solution at scan rate of 3 mV/s in an open electrochemical cell.

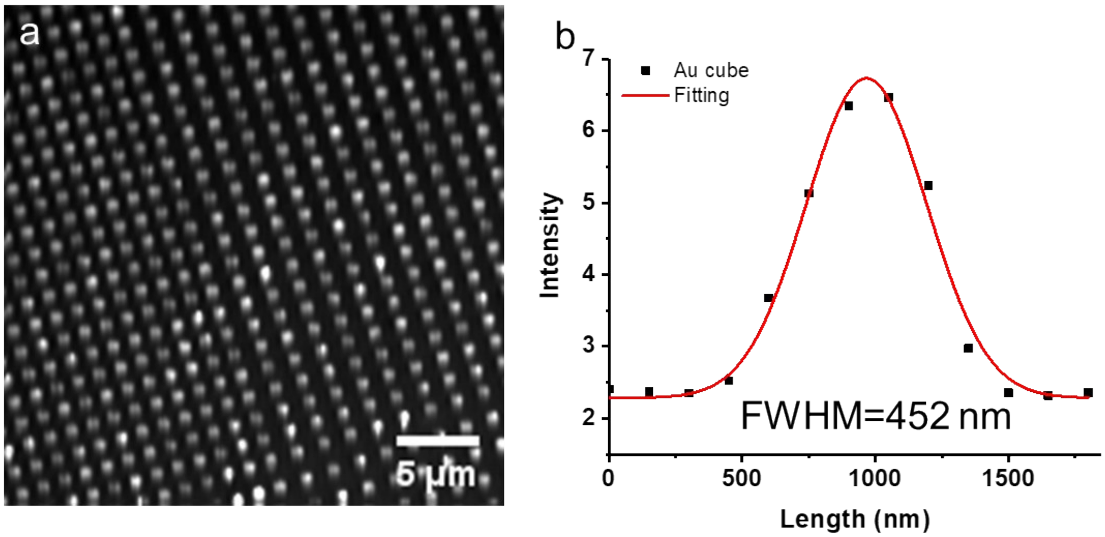

Figure S3. (a) Photothermal imaging of 500 nm Au nanocubes. (b) A cross section line of a representative nanocube.

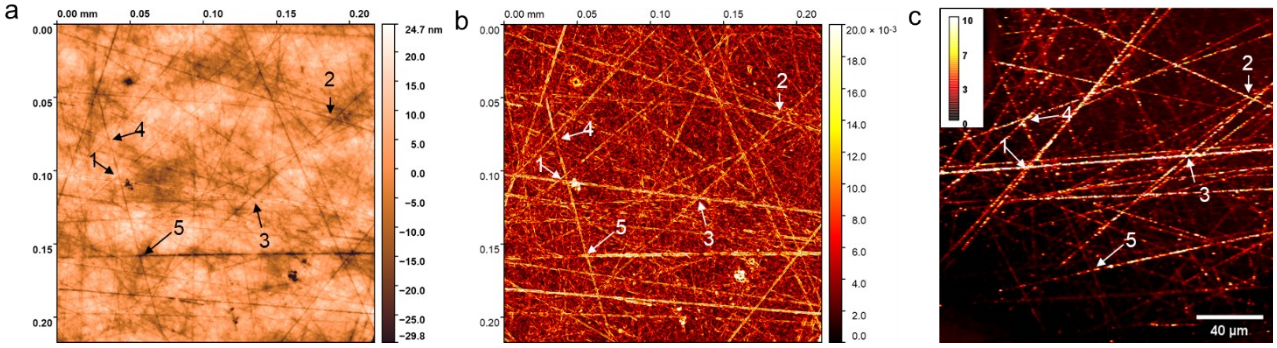

Figure S4. Typical topography of a polished Pt electrode and corresponding photothermal image. (a) Topography image of the Pt electrode. (b) The corresponding local slope image from the topography image. (c) The corresponding photothermal image. The arrows indicate the corresponding points of intersection.

The local slope image can reflect the relative roughness of the Pt surface. A larger local slope indicates more roughness. The photothermal signal from a rough area (e.g. scratches) is much stronger than that of a smooth area.

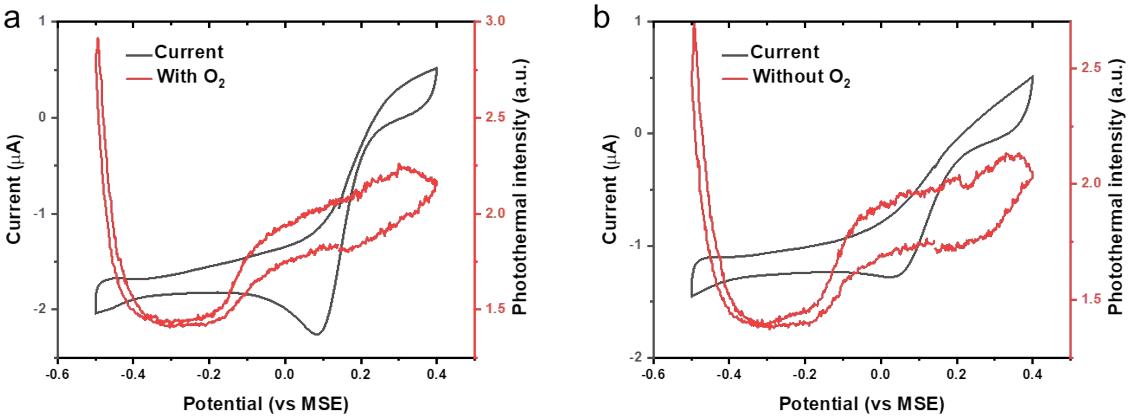

Figure S5. EPRM monitoring during the CV of Pt in H_2SO_4 solution with/without dissolved O_2. (a) CV curve and corresponding average photothermal intensity during CV measurement of the Pt electrode in a 0.5 M H_2SO_4 solution at a scan rate of 3 mV/s. (b) CV curve and corresponding average photothermal intensity during CV measurement of the Pt electrode in a deoxygenated 0.5 M H_2SO_4 solution at a scan rate of 3 mV/s.

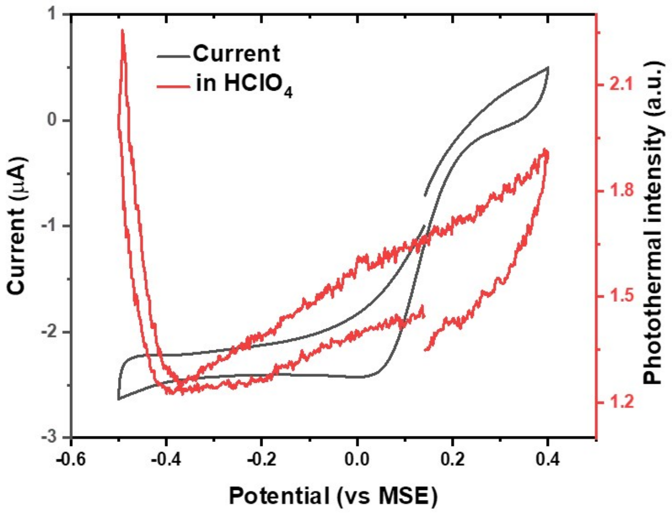

Figure S6. CV curve and corresponding average photothermal intensity during CV measurement of the Pt electrode in a 1 M $HClO_4$ solution at a scan rate of 3 mV/s.

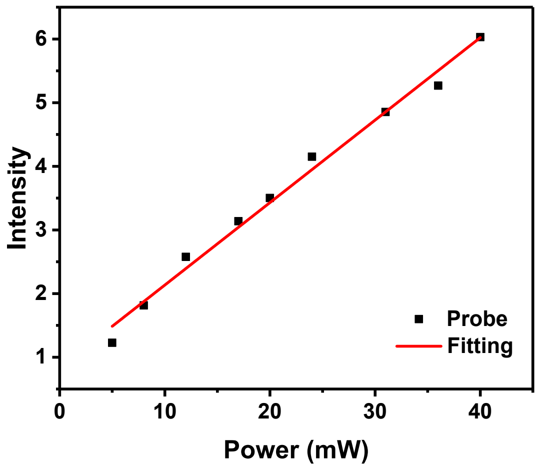

Figure S7. The dependence of the photothermal intensity on the probe laser power

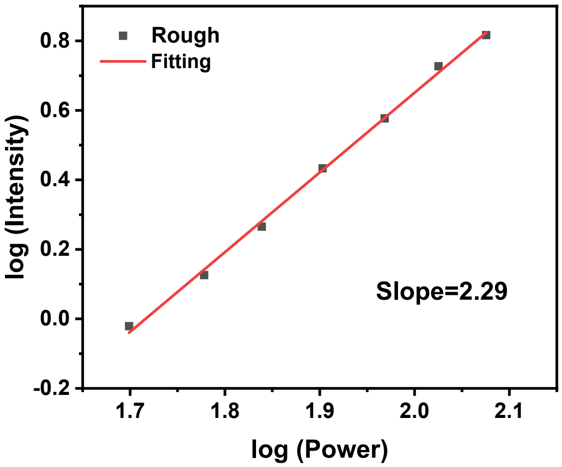

Figure S8. The dependence of the photothermal intensity on the pump laser power in the rough area.

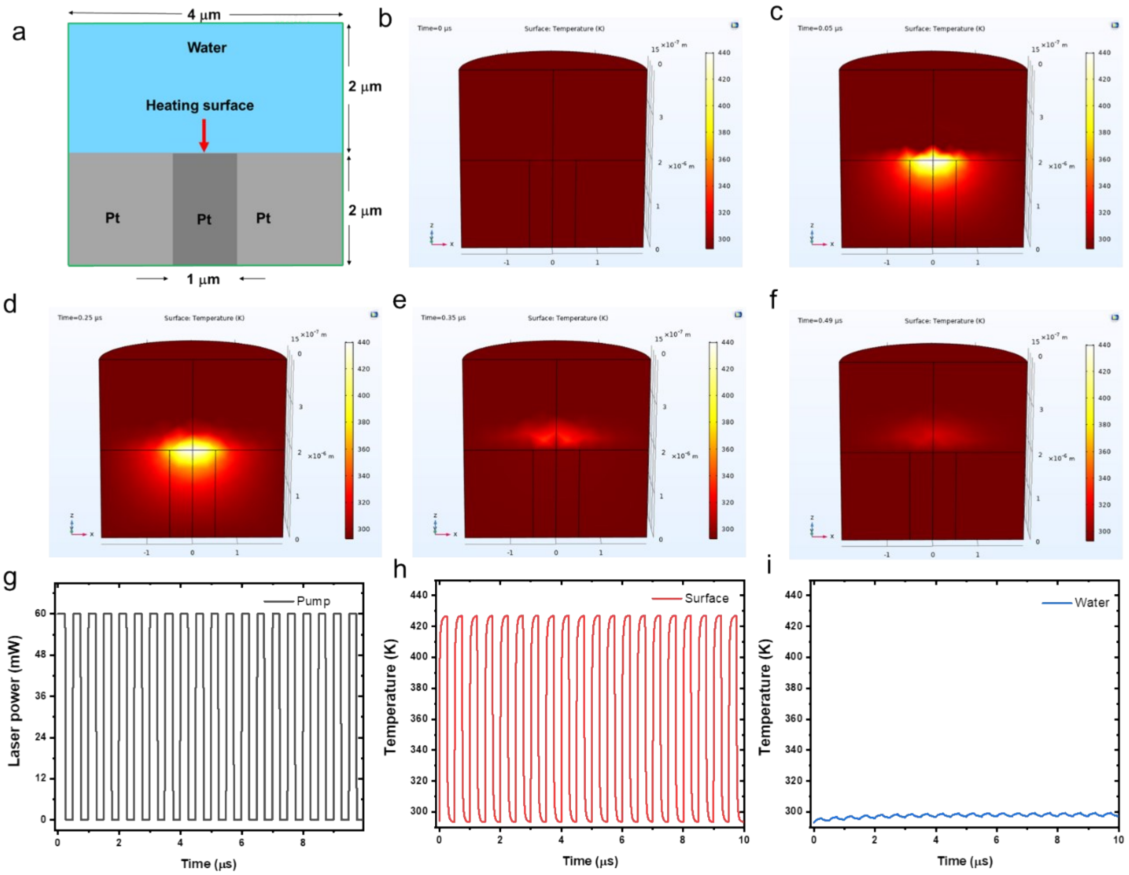

Supporting information 1. Local temperature change at the Pt electrode

Figure S9. COMSOL simulation of local temperature changes. (a) Geometric configuration used in the simulation by COMSOL. (b-d) The local temperature distribution at 0 μs, 0.05 μs, 0.25 μs, 0.35 μs, and 0.49 μs, respectively. (g) The waveform of the modulated pump laser. (h) The time dependent temperature of the illuminated Pt surface. (i) The time dependent temperature of water.

The temperature simulation was carried out using COMSOL Multiphysics software. Figure S9a shows geometric configuration used in the simulation. Heat transfer in solids was chosen. The 60 mW 1040 nm laser with a 2 MHz modulation illuminated at the center of the Pt surface as a heat source. The illuminated area is 1 μm. The reflectance of Pt at 1040 nm is 0.8.[1] The green lines in Figure S9a show boundaries of a constant temperature (initial temperature, 293K). Figure S9b-d present the time-lapse simulation results of the temperature distribution in the first cycle. When laser turns on, the temperature of illumination Pt surface quickly increases, and then heat transfer to water. When laser turns off, heat continually transfer from Pt to water. The Pt surface quickly recovers back to initial temperature, and the temperature of water slightly increases. Figure 9g shows the 2 MHz square waveform modulated pump laser. As shown in figure 9h, the temperature of illuminated surface quickly and then gradually increases to 426 K when laser turns on. When laser turns off, the temperature of laser irradiation surface immediately drops back to

293 K less than 0.1 μs. The surface recover to initial temperature before the next laser on period. In figure S9i, we can also observe the temperature of water periodically rises and decays with the modulated laser irradiation. The maximum temperature of water is 300 K and the final temperature of local water is 297 K after 10 μs laser illumination.

Reference

1. W. S. M. Werner, K. Glantschnig and C. Ambrosch-Draxl, *J. Phys. Chem. Ref. Data*, 2009, **38**, 1013-1092.
